# Overcoming cisplatin resistance by targeting the MTDH-PTEN interaction in ovarian cancer with sera derived from rats exposed to Guizhi Fuling wan extract

**DOI:** 10.1186/s12906-020-2825-9

**Published:** 2020-02-17

**Authors:** Li Han, Xueyun Cao, Zhong Chen, Xiaojuan Guo, Lei Yang, Yubing Zhou, Hua Bian

**Affiliations:** 1 Zhang Zhongjing College of Chinese Medicine, Nanyang Institute of Technology, Nanyang, 473004 China; 2 Henan Key Laboratory of Zhang Zhongjing Formulae and Herbs for Immunoregulation, Nanyang Institute of Technology, Nanyang, 473004 China; 3 College of Pharmaceutical Science, Soochow University, Suzhou, 215123 China; 4 Department of Pharmacy, the First Affiliated Hospital of Zhengzhou University, Zhengzhou, 450052 China

**Keywords:** Guizhi Fuling wan, Ovarian Cancer, Chemotherapy resistance, MTDH, PTEN

## Abstract

**Background:**

The well-known traditional Chinese herbal formula Guizhi Fuling Wan (GFW) was recently reported to improve the curative effects of chemotherapy for ovarian cancer with few clinical side effects. The present study aimed to investigate the reversal mechanism of sera derived from rats exposed to Guizhi Fuling Wan extract (GFWE) in cisplatin-resistant human ovarian cancer SKOV3/DDP cells; the proteins examined included phosphatase and tensin homolog (PTEN) and metadherin (MTDH), and the possible protein interaction between PTEN and MTDH was explored.

**Methods:**

GFWE was administered to healthy Wistar rats, and the sera were collected after five days. The PubMed and CNKI databases were searched for literature on the bioactive blood components in the sera. The systemsDock website was used to predict potential PTEN/MTDH interactions with the compounds. RT-qPCR, western blotting, and immunofluorescence analyses were used to analyze the mRNA and protein levels of MTDH and PTEN. Laser confocal microscopy and coimmunoprecipitation (co-IP) were used to analyze the colocalization and interaction between MTDH and PTEN.

**Results:**

Sixteen bioactive compounds were identified in GFWE sera after searching the PubMed and CNKI databases. The systemsDock website predicted the potential PTEN/MTDH interactions with the compounds. RT-qPCR, western blotting, and immunofluorescence analyses showed decreased MTDH expression and increased PTEN expression in the sera. Laser confocal microscopy images and coimmunoprecipitation (co-IP) analyses demonstrated that a colocalization and interaction occurred between MTDH and PTEN, and the addition of the sera changed the interaction status.

**Conclusions:**

GFWE restored sensitivity to cisplatin by inhibiting MTDH expression, inducing PTEN expression, and improving the interaction between MTDH and PTEN in SKOV3/DDP cells, and these proteins and their interaction may serve as potential targets for cancer treatment. The sera may represent a new source of anticancer compounds that could help to manage chemoresistance more efficiently and safely.

## Background

Ovarian cancer (OC) is the seventh most common cancer and the eighth cause of cancer-related death among women with a range of 30–50% of the age-standardized 5-year net survival rate [1, 2]. OC is generally advanced when it is diagnosed because there are typically no apparent symptoms during the early stages. The standard of care usually consists of platin-based drugs combined with nonplatins, such as paclitaxel or docetaxel, for advanced-stage OC patients or high-risk surgical candidates [3]. More than 70% of patients who initially respond to chemotherapy, including platins and nonplatins, ultimately have recurrence and develop chemoresistance [4]. To this end, exploring the molecular mechanism associated with chemoresistance and finding strategies to improve the therapeutic effect of OC is exceptionally urgent for researchers.

The function of PI3K/Akt/mTOR pathway in OC chemoresistance has received increased attention across a number of disciplines in recent years [5, 6]. Inactivation of PI3K/Akt/mTOR signaling is an effective strategy to re-sensitize chemoresistant OC to chemotherapeutic drugs. Although promising results in several phase I and II studies suggest that mTOR inhibitors may overcome chemoresistance for OC patients, the clinical practice is limited by the toxicity profile of mTOR inhibitors [7]. More broadly, researches are needed to explore other possible targeting as new therapeutic strategies. The role of metadherin (MTDH), also known as astrocyte elevated gene-1 (AEG-1) or lysine-rich CEACAM1 coisolated (LYRIC), in cancer chemoresistance is noted in a number of studies, and downstream molecular mechanisms implicated in chemoresistance include NF-kB, AMPK/ATG5, P13K/AKT/mTOR, and Wnt/β-catenin signaling [8–12]. MTDH overexpression was strongly associated with poor prognosis and cisplatin resistance in advanced serous OC [13], while a loss of MTDH could lead to the inhibition of cell migration and invasion and restore sensitivity to chemotherapeutics. Therefore, inhibition of MTDH could be a useful therapeutic target for reversing the chemoresistance of OC.

Phosphatase and tensin homolog (PTEN), a natural inhibitor of the PI3K/AKT pathway, is one of the most commonly lost tumor suppressors in human cancer. Recently, researchers have shown an increased interest in the role of PTEN in chemoresistance. It was reported that up to 50% of castration-resistant prostate cancer cases are estimated to have a loss of PTEN [14]. There are similarities between the loss of PTEN and chemoresistance in other diseases, such as colorectal cancer, glioma, and non-small-cell lung cancer [15–17]. Previous studies have also shown that low expression or loss of PTEN was a significant risk factor for the chemoresistance of OC patients [18, 19]. Inducing PTEN also plays a critical role in restoring the sensitivity of anticancer drugs to OC. What remains unknown, however, is whether MTDH interacts with PTEN and whether the interaction serves a role in chemoresistance. Modulation of protein-protein interactions (PPIs), which are the physical contacts of high specificity established between two or more protein molecules as a result of biochemical events, is receiving increasing attention by the scientific community [20]. It is known that disruptions of the normal patterns of PPIs and multiprotein complexes can be causative or indicative of a disease state in humans. For instance, MTDH can interact with CREB-binding protein (CBP) and then prevent its ubiquitin-mediated degradation, licensing the transcriptional activation of TWIST1 [21], which has been shown to contribute to OC cisplatin resistance [22].

Guizhi Fuling Wan (GFW; Gyejibokryeong-hwan in Korean and Keishi-bukuryo-gan in Japanese) is a well-known Chinese classic herbal formula, which is a combination of herbs used in Chinese herbology for greater efficiency than individual herbs, and has been used extensively throughout Asia for the treatment of blood stasis; this formula has been shown to be very safe and effective with few harmful side effects and was established more than 2000 years ago. GFW was developed by Zhang Zhongjing (150–219 A.D.) at the end of the Eastern Han Dynasty. Numerous studies have also confirmed the safety of GFW for genotoxic effects [23], adverse reactions [24], and subchronic toxicity [25]. Several researchers have reported that GFW has various therapeutic effects on conditions, such as climacteric syndrome [26], arteriole vasodilation [27], inflammatory skin disorders [28], endometriosis [29], diabetes-mellitus-induced neuropathology [30], and atherosclerosis [31]. Recent evidence also suggests that GFW has an antitumor effect on bladder cancer [32] and breast cancer [33]. Chinese clinicians have shown that GFW could improve the curative effect of chemotherapy for cervical carcinoma [34] and oophoroma [35] with few clinical side effects; however, the underlying mechanism is unclear. We previously demonstrated that GFW could sensitize cisplatin-resistant SKOV3/DDP cells by inhibiting the protein level and function of P-glycoprotein (P-gp) and by inactivating the PI3K/AKT/mTOR pathway; in the resistant SKOV3/DDP xenograft tumour mice, GFW could enhance anticancer efficacy of cisplatin and paclitaxel without any mouse deaths during a 21-day administration at a dose of 4 g·kg^− 1^·d^− 1^ [36]. We have also shown that paeonol, one of the bioactive constituents of GFW, has an effect on the inhibition of MTDH and the induction of PTEN in SKOV3/DDP cells [37].

In light of the knowledge of the above facts, we evaluated whether GFW inhibits MTDH and induces PTEN to address the chemoresistance issues in OC treatment. We hypothesized that MTDH would interact with PTEN, and the interaction would contribute to chemoresistance. We then further explored the role of GFW in disturbing the interaction between MTDH and PTEN in SKOV3/DDP cells; this study thus presents a promising strategy for the clinical practice of GFW in restoring sensitized cisplatin-resistant cells.

## Methods

### Plant materials and preparation of the GFWE

GFW consists of five herbs, and the mixed proportion of herbs is illustrated in Table 1. All botanical names can be checked and validated using http://www.theplantlist.org. The herbs used in this study were purchased from Nanyang Zhongjing Baixin Medical Technology Co., Ltd. (Nanyang, China). The plant materials were authenticated based on their morphological characteristics by Prof. Chaoyun Zhang, a plant taxonomist from the Zhang Zhongjing College of Chinese Medicine, Nanyang Institute of Technology, where the voucher specimens were deposited (Additional file 1). Assurance of quality control for all the materials was validated according to the Chinese Pharmacopoeia (China Pharmacopoeia Committee, 2015).

**Table 1:** The composition and proportion of herbs in GFW

Botanical name	Herbal name	Chinese name	Voucher number	Dosage
*Cinnamomum cassia* (L.) J. Presl	*Ramulus Cinnamomi cassia*	Gui-Zhi	170,801	15 g
*Smilax glabra Roxb.*	*Sclerotium Poriae Cocos*	Fu-Ling	170,901	15 g
*Prunus persica* (L.) Batsch	*Semen Pruni Persicae*	Tao-Ren	170,801	15 g
*Paeonia suffruticosa var. papaveracea* (Andrews) A. Kern.	*Cortex Radicis Moutan*	Mu-Dan-Pi	170,801	15 g
*Paeonia lactiflora* Pall.	*Radix Albus Paeoniae Lactiflorae*	Shao-Yao	170,601	15 g

GFWE was prepared following the process as stated in the Chinese Pharmacopoeia as previously described [36, 38]. Briefly, the water-soluble ingredients, liposoluble ingredients and volatile oil of GFWE were extracted, and the extracts were combined uniformly. The concentration was adjusted to 1.5 g/mL corresponding to the crude herbs. The mixed solution was used for animal studies and preparing the medicated rat sera.

### Preparation of rat sera after GFWE exposure

The rat sera after GFWE exposure were made according to the published protocols described previously with a slight modification [36]. Briefly, 60 healthy Wistar female rats, aged between 6 and 8 weeks old and weighing 220~250 g, were randomly divided into four groups with 15 rats per group: the control group administered normal saline by gavage and the low-dose (LD) GFWE group, middle-dose (MD) GFWE group and high-dose (HD) GFWE group administered the mixed GFWE solution by gavage at dosages of 4 g·kg^− 1^·d^− 1^, 8 g·kg^− 1^·d^− 1^, and 16 g·kg^− 1^·d^− 1^, respectively, based on clinical dosage of GFW. The animals were supplied by the Henan Experimental Animal Center (Zhengzhou, China). This study was carried out in accordance with the accepted standards of humane animal care as outlined in ethical guidelines on the care and use of laboratory animals in an air-conditioned room with a controlled temperature of 22 ± 2 °C, a humidity level of 45 to 65%, and a 12−/12-h light/dark cycle. All animal procedures were conducted with protocol approval from the Ethics Committee of Nanyang Institute of Technology (Nanyang, China), and all efforts were made to minimize animal suffering. After five days of administration, the rats were anesthetized with 1.5% isoflurane in an anesthesia mask, and the blood was collected from the jugular vein; all blood samples from the same group from the experimental rats were combined into one tube to eliminate individual variability. No rats died, and no body weight loss was observed during the 5-day administration of the 3 doses. At the end of the study, the animals were euthanized with 30% CO_2_. The samples of different groups were centrifuged, and the collected sera were filtered through a Millipore filter for sterilization, aliquoted into 1-mL Eppendorf tubes and preserved at − 80 °C for future use.

### Blood bioactive ingredients identification and molecular docking

As GFW is widely used in China, Japan, and Korea, the bioactive compounds in the sera containing GFWE have already been demonstrated elsewhere. After searching the PubMed and CNKI databases to collect the literature on the blood bioactive ingredients of the sera, the systemsDock website (http://systemsdock.unit.oist.jp, Version 2.0) [39], a web server for compound-protein docking simulation with good prediction accuracy, was used to predict potential PTEN/MTDH interactions with the compounds. The PI3K/AKT inhibitor LY294002, which has been shown to modulate PTEN/MTDH [40, 41], was used as a positive compound when the docking simulation was applied.

### Cell culture and cytotoxicity assay

The human OC SKOV3 cell line and its cisplatin-resistant SKOV3/DDP cell line were obtained from the Chinese Academy of Medical Sciences and Peking Union Medical College. The cells were treated as previously described [36].

The ability of the medicated rat sera containing GFWE to potentiate cisplatin cytotoxicity was evaluated in SKOV3/DDP and SKOV3 cells using the xCELLigence Real-Time Cell Analysis (RTCA) S16 instrument (ACEA Biosciences, San Diego, CA, USA), which is an impedance-based platform for monitoring cell viability; the instrument was placed in a humidified incubator at 37 °C with 5% CO_2_. Cytotoxicity assays were performed using E-plate 16 (ACEA Biosciences, San Diego, CA, USA), which was inserted into the platform during experiments. The cells were harvested in the exponential growth phase and seeded into the plate at a density of 5.0 × 10^3^ cells per well with fetal bovine serum (FBS)-free RPMI 1640 medium. Six hours after seeding, LD sera plus cisplatin or cisplatin alone at final concentrations of 16.7, 8.3, 5.0 and 2.5 μg/ml were added to the culture. The sera volume ratio for incubation was 10%. All incubation was performed at a final volume of 200 μL. The impedance was recorded at 15-min intervals. The amount of growth area covered in the E-plate 16 due to cell adhesion was represented as the cell index (CI). A high CI indicates high levels of cell adhesion and high cell viability and vice versa. The inhibition growth by 50% (IC_50_ value) was calculated based on the CIs.

### RT-qPCR analysis

To analyze the mRNA expression, SKOV3/DDP cells were seeded into 12-well plates at a density of 2.0 × 10^5^ cells per well and were treated with medicated rat control sera, LD, MD, HD sera or LY294002 (10 μmol·L^− 1^; MedchemExpress, USA) for 48 h. Total RNA was extracted using the UNlQ-10 Column TRIzol Total RNA Isolation Kit (Sangon Biotech, China) according to the manufacturer’s instructions. Extracted RNA was assessed for purity and quantity using a Nanodrop 2000 Spectrophotometer (Thermo Fisher, USA). A total of 500 ng of total RNA was used for cDNA synthesis using the RevertAid First Strand cDNA Synthesis Kit (Thermo Fisher Scientific Inc., USA). A quantitative comparison of the mRNA levels was performed using FastStart Universal SYBR Green Master with Rox (Merck KGaA, Germany) in an Applied Biosystems ViiA7 Real-Time PCR System by the manufacturer’s recommended thermal cycling conditions. The forward and reverse primers for amplifying MTDH are as follows: 5′-CCTGGCCTTGCTGAAGAATC-3′ and 5′-GGCTGCTTTGCTGTTACACT-3′, and the length of the amplified fragment was 150 bps. The forward and reverse primers for amplifying PTEN were as follows: 5′-ATGTTCAGTGGCGGAACTTG-3′ and 5′-CACACAGGTAACGGCTGAGG-3′, and the length of the amplified fragment was 137 bps. A 196-bp fragment of GAPDH was used as the internal control and was amplified with the following forward and reverse primers: 5′-GAGTCAACGGATTTGGTCGT-3′ and 5′-GACAAGCTTCCCGTTCTCAG-3′. The relative gene expression analysis was performed using the 2^−ΔΔCt^ method as described previously [42].

### Western blot assay

Western blotting analyses of the MTDH and PTEN proteins were performed using a slight modification of the method described previously [43]. Briefly, SKOV3/DDP cells were treated as above for 48 h, and then the cells were lysed using a Minute™ Total Protein Extraction Kit (Invent Biotechnologies, USA). The protein concentrations were determined using the Pierce Rapid Gold BCA Protein Assay Kit (Thermo Fisher, USA). A total of 20 μg of protein per sample was electrophoresed via sodium dodecyl sulfate-polyacrylamide gel electrophoresis (SDS-PAGE) and transferred onto PVDF membranes (Millipore, USA), which were then blocked with 5% skim milk for 1 h at room temperature (RT). The membranes were then incubated with a rabbit polyclonal antibody against MTDH (1:600 dilution; Proteintech Group, China), a mouse monoclonal antibody against PTEN (1:1000 dilution; Proteintech Group, China), and a mouse monoclonal antibody against GAPDH (1:4000 dilution; Proteintech Group, China) at 4 °C overnight. After three washes with TBST, the primary antibodies were then detected using species-matched secondary antibodies (Proteintech Group, China) with a 1.5 h incubation at RT. The membranes were incubated with Pierce ECL Western Blotting Substrate (Thermo Fisher, USA) for 5 min in the dark. The bands were detected using the Tanon 5200 Blots Imaging System (Tanon Science & Technology Co., Ltd., China).

### Immunofluorescence and laser scanning confocal microscopy

SKOV3/DDP cells in the different groups were seeded in 20 mm diameter NEST confocal glass-bottom cell culture dishes at 37 °C and 5% CO_2_ for 48 h. The cells were then fixed with 4% paraformaldehyde for 5 min and kept stable in 0.5% Triton X-100 for 10 min to rupture the cell membranes. Following three PBS washes, nonspecific antigen-binding sites were blocked with 5% goat serum for 1 h at RT. The cells were then incubated with a rabbit polyclonal antibody (pAb) against MTDH (1:300 dilution; Proteintech Group, China) and a mouse monoclonal antibody (mAb) against PTEN (1:500 dilution; Proteintech Group, China) overnight at 4 °C. After washing, the cells were incubated with the corresponding species-matched Alexa Fluor-488/647-conjugated secondary antibodies (Proteintech Group, China) for 1 h at RT. After washing, the nuclei were stained with DAPI for 2 min, and the slides were washed with PBS. The dishes were kept from light before being observed with a fluorescence microscope. The observation was made using a Zeiss LSM800 Laser Scanning Confocal Microscope (Carl Zeiss AG, Germany). For the analysis of protein level, images were acquired using the 40× Plan-Apochromat objective (numerical aperture, NA: 0.95), and mean fluorescence intensities (MFI) were measured after background subtraction with the graphics module of Zeiss Zen software (version 2.3). For the analysis of cellular protein localization, images were acquired using the 63× Plan-Apochromat oil objective (numerical aperture, NA: 1.40) with Airyscan, and the colocalization coefficients of MTDH/PTEN were analyzed by the Confocal Topography module of Zeiss Zen software.

### Coimmunoprecipitation (co-IP) assay

The SKOV3/DDP cells in different groups as above were seeded in a Corning 25 cm^2^ Cell Culture Flask at 37 °C and 5% CO_2_ for 48 h. SureBeads™ Starter Kit Protein A/G Beads (Bio-Rad Laboratories, Inc., USA) were precleaned with 1.5 μg of pAb-MTDH or pAb-IgG for 20 min at RT before co-IP. The cells were lysed using a Minute™ Total Protein Extraction Kit (Invent Biotechnologies, Inc., USA), and the protein concentrations were determined using a Pierce Rapid Gold BCA Protein Assay Kit (Thermo Fisher, USA). A total of 300 μg protein per sample was incubated with the beads and rotated for 1 h at RT. After washing 3 times with PBS buffer containing 0.1% Tween-20, the beads were incubated with Laemmli Sample Buffer containing β-mercaptoethanol for 10 min at 70 °C. After the beads were magnetized, the eluent was boiled for 5 min at 95 °C before being loading onto an SDS-PAGE gel and performing western blotting analysis with the mAb-PTEN.

### Statistics

Data were expressed as the mean ± SD of at least three independent experiments. Statistical evaluation of the data was performed using one-way ANOVA with Tukey’s multiple comparisons test. The colocalization of MTDH with PTEN was investigated using both Pearson’s correlation coefficient and Manders’ colocalization coefficient. Statistical analysis was performed with GraphPad Prism version 6.01 for Windows. Differences were considered to be statistically significant for *P* < 0.05.

## Results

### The potential modulating effect of GFWE on PTEN/MTDH

**Fig. 1:**
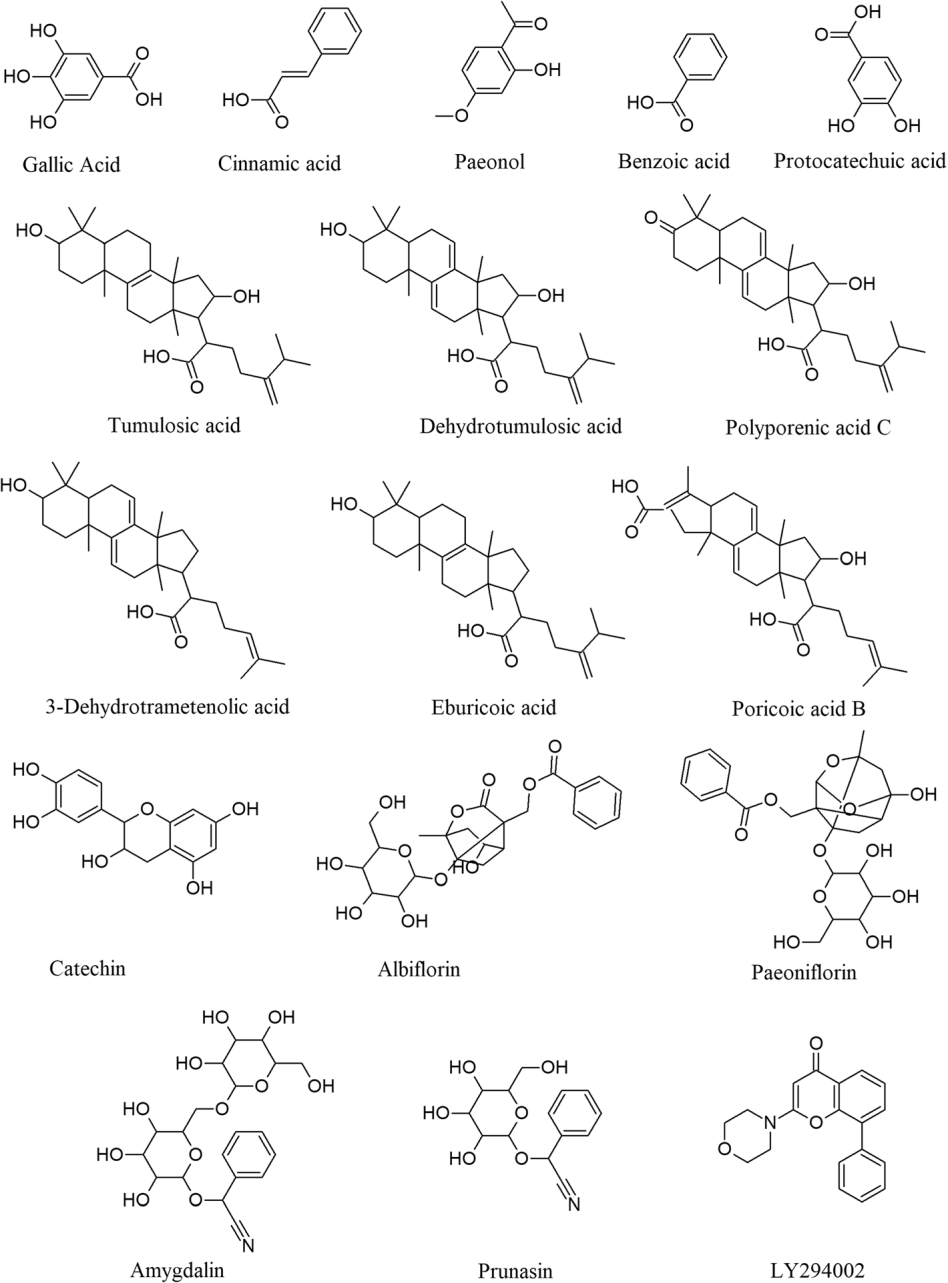
Main active ingredients from rat sera containing GFWE and the positive control compound LY294002

**Fig. 2:**
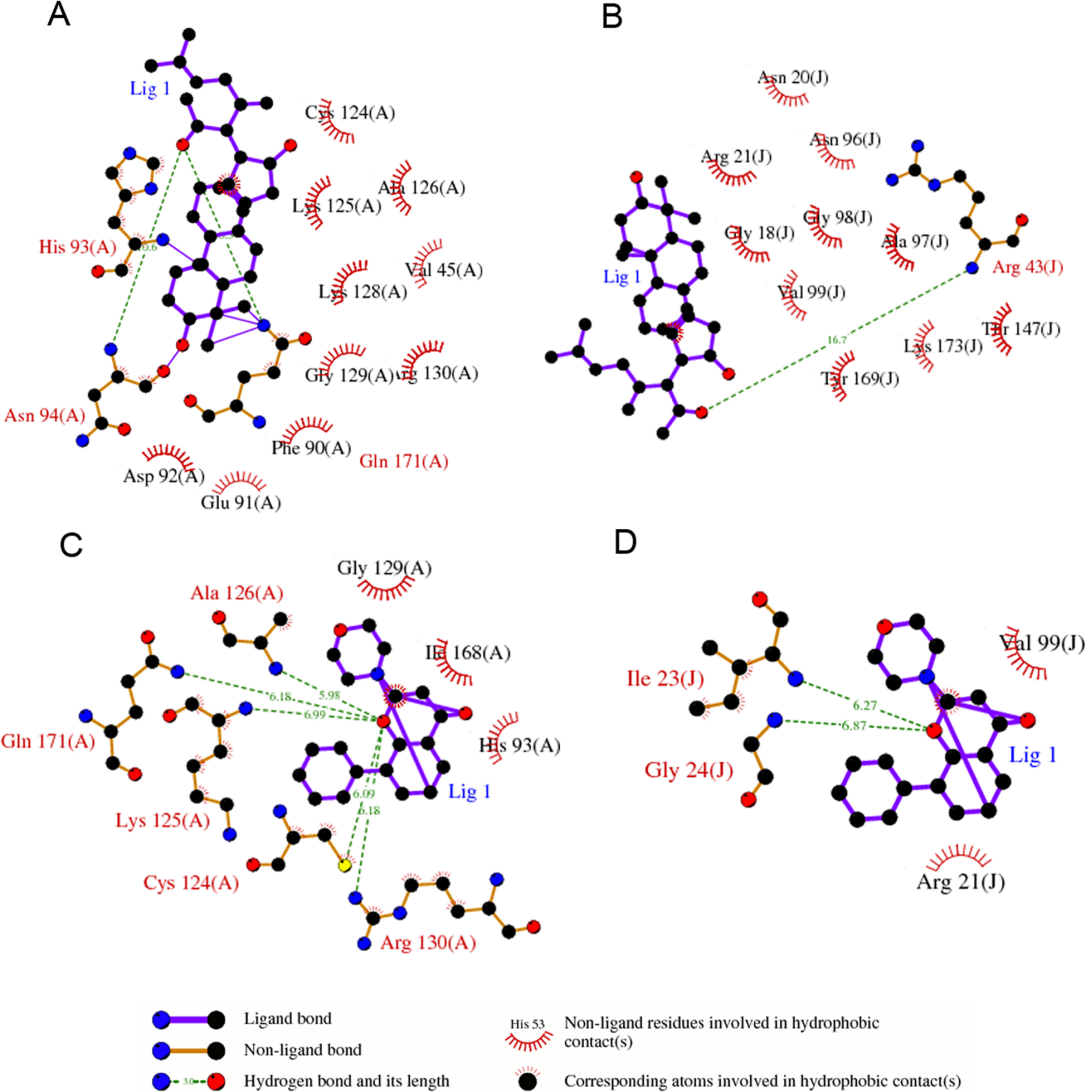
Protein Ligand interaction with LY294002 and 3-dehydrotrametenolic acid. The systemsDock website was used to predict the possible PTEN/MTDH protein ligand interaction with the compounds. **a**. PTEN protein ligands with 3-dehydrotrametenolic acid, **b**. MTDH protein ligands with 3-dehydrotrametenolic acid, **c**. PTEN protein ligands with LY294002, **d**. MTDH protein ligands with LY294002

The PubMed and CNKI databases were searched for the UHPLC-MS/MS, UPLC/Q-TOF-MS/MS, or ^1^H NMR with UPLC-MS methods for detecting sera bioactive ingredients after rats were administered GFW orally, and 16 bioactive compounds were identified in the sera (Fig. 1): cinnamic acid, paeonol, benzoic acid, prunasin, albiflorin, protocatechuic acid, amygdalin, paeoniflorin, gallic acid, catechin, tumulosic acid, polyporenic acid C, dehydrotumulosic acid, poricoic acid B, eburicoic acid, and 3-dehydrotrametenolic acid [44–47]. Three of the 16 bioactive compounds, paeonol, gallic acid and paeoniflorin, were also identified in the sera samples using HPLC/QqQ MS in our previous study [36]. The molecular docking results showed that 14 of 16 compounds have a higher binding activity with PTEN (PDB ID: 1D5R)/MTDH (PDB ID: 1H5Q) compared to LY294002. 3-Dehydrotrametenolic acid has the highest docking score with PTEN/MTDH, and cinnamic acid has the lowest docking score with PTEN/MTDH. The docking score of paeonol with PTEN/MTDH was slightly weaker than that of LY294002, suggesting that most of the blood bioactive ingredients of GFWE have a potential modulating effect on PTEN/MTDH (Table 2). Further analysis by systemsDock showed that the interactions between the docking scores of PTEN/MTDH and the tested compounds may be related to the number of interacting residues, e.g., PTEN has 13 residues (Arg A 130, His A 93, Lys A 125, Cys A 124, Gln A 171, Ala A 126, Gly A 129, Asn A 94, Lys A 128, Asp A 92, Phe A 90, Val A 45, and Glu A 91) that interact with 3-dehydrotrametenolic, but 8 residues (Arg A 130, His A 93, Lys A 125, Cys A 124, Gln A 171, Ala A 126, Gly A 129, and Ile A 168) interacted with LY294002; MTDH has 11 residues (Ala J 97, Gly J 98, Val J 99, Thr J 147, Asn J 96, Gly J 188, Asn J 20, Arg J 21, Arg J 43, Tyr J 169, and Lys J 173) that interact with 3-dehydrotrametenolic, but 4 residues (Arg J 21, Ile J 23, Val J 99, and Gly J 24) interacted with LY294002 (Fig. 2).

**Table 2:** Docking scores of blood bioactive ingredients of GFWE and LY294002 to PTEN/MTDH

Compounds	Docking Scores (pKd/pKi)	
	MTDH	PTEN
Cinnamic acid	3.06	2.82
Paeonol	3.78	3.90
LY294002	4.47	4.02
Benzoic acid	5.67	4.96
Prunasin	5.73	5.59
Albiflorin	6.14	5.98
Protocatechuic acid	6.14	5.18
Amygdalin	6.18	5.33
Paeoniflorin	6.26	6.21
Gallic acid	6.42	5.43
Catechin	6.99	6.16
Tumulosic acid	7.95	7.67
Polyporenic acid C	8.01	7.68
Dehydrotumulosic acid	8.04	6.21
Poricoic acid B	8.16	7.46
Eburicoic acid	8.16	8.02
3-Dehydrotrametenolic acid	8.17	8.08

Note: The docking score represents the weak to strong binding activity ranging from 0 to 10

### Sera containing GFWE strengthen the potency of cisplatin

xCELLigence RTCA showed that the IC_50_ of SKOV3/DDP and SKOV3 cells to cisplatin was 15.53 ± 0.11 mg·L^− 1^ and 3.40 ± 0.14 mg·L^− 1^, respectively. The LD, MD and HD sera alone had a less than 30% growth inhibition effect on SKOV3 and SKOV3/DDP cells, and the LD sera alone had the lowest inhibition rate (< 5%). Treatment with the LD sera induced a significant reduction in the IC_50_ of cisplatin against SKOV3/DDP cells to 3.04 ± 0.18 mg·L^− 1^. However, no such activity was observed in SKOV3 cells (3.40 ± 0.14 vs. 3.28 ± 0.21 mg·L^− 1^, *P* > 0.05). These findings indicate that the sera containing GFWE enhance the potency of cisplatin against SKOV3/DDP cells, whereas it had little effect on SKOV3 cells, supporting the notion that the sera containing GFWE reverse the resistance of SKOV3/DDP cells. These results are similar to those of our previously conducted study using CCK-8 kits [36].

### Sera containing GFWE modulate MTDH/PTEN at the mRNA level

**Fig. 3:**
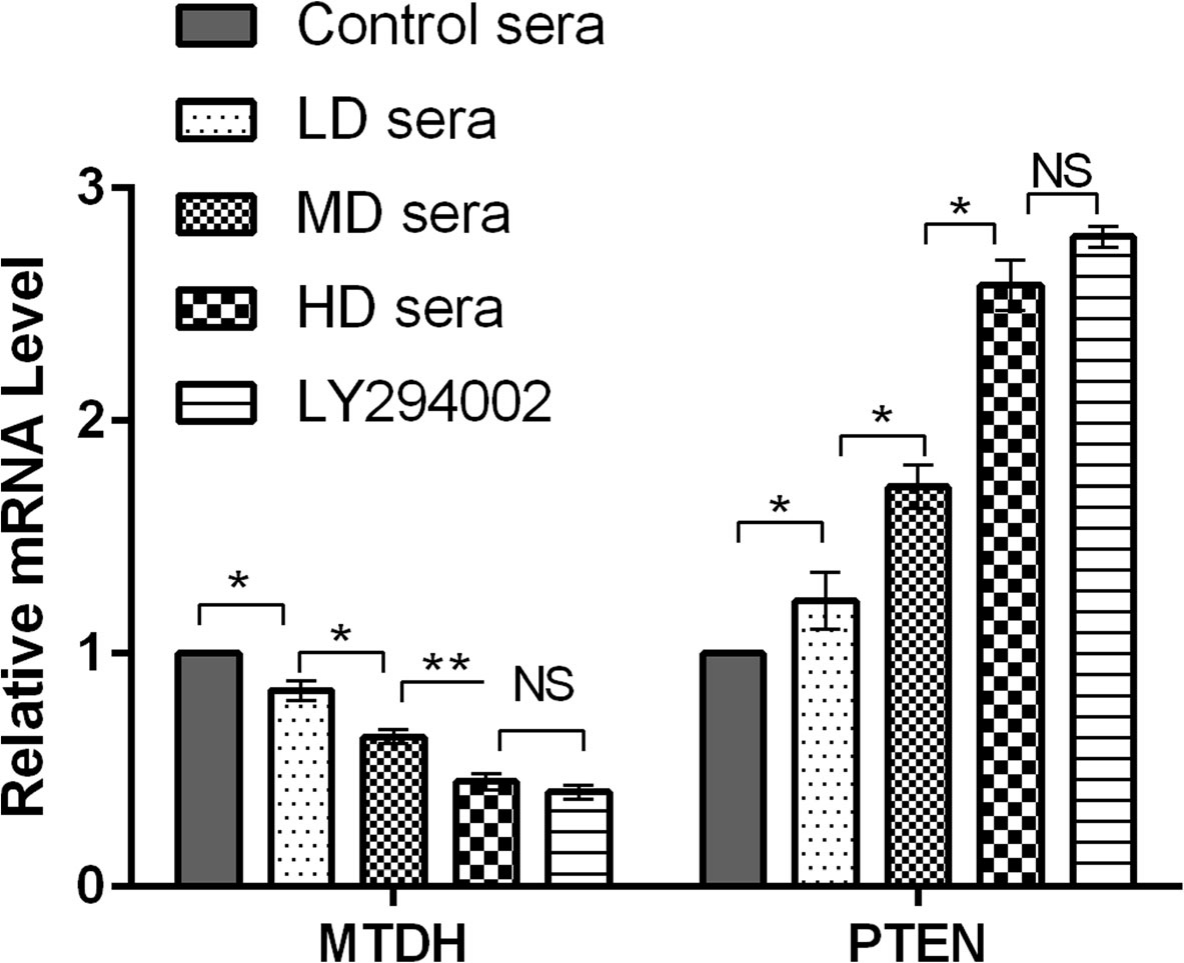
Relative quantification of MTDH and PTEN mRNA in SKOV3/DDP cells. A 2^−ΔΔCt^ method was used to analyze the mRNA expression changes after treatment with sera containing GFWE or LY294002, and GAPDH was used as an internal standard. LD sera = low-dose sera containing GFWE, MD sera = middle-dose sera containing GFWE, HD sera = high-dose sera containing GFWE. Error bars represent the SD. *P < 0.05, **P < 0.01

To examine whether MTDH/PTEN was downregulated by the sera containing GFWE at the mRNA level, we analyzed MTDH and PTEN mRNA expression by RT-qPCR. After 48 h of sera treatment, the expression of PTEN mRNA in SKOV3/DDP cells increased in a dose-effect pattern. The expression of MTDH mRNA in SKOV3/DDP cells was significantly inhibited with increasing sera dose (*P* < 0.05, *P* < 0.01). Compared to the HD sera group, the LY294002 group showed a similar inhibition of MTDH mRNA and induction of PTEN mRNA expression (Fig. 3). Nevertheless, these data indicated that the sera modulate MTDH/PTEN at the mRNA level.

### Sera containing GFWE modulate MTDH/PTEN at the protein level

**Fig. 4:**
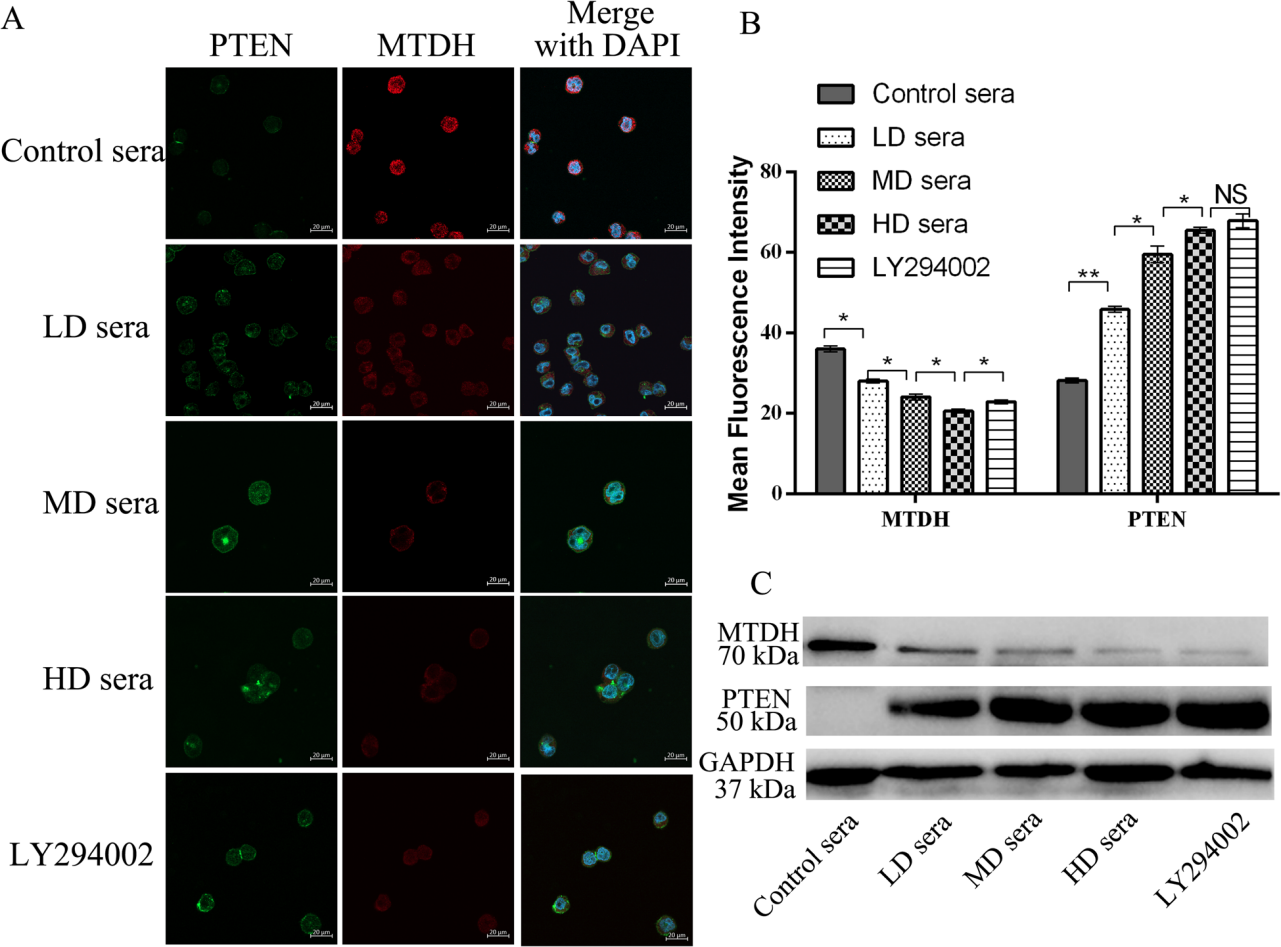
The protein expression changes of MTDH and PTEN in SKOV3/DDP cells treated with the sera containing GFWE. (**a**) Representative images for MTDH and PTEN investigated. All images were obtained using a 40× Plan-Apochromat objective of the LSM800 confocal microscope. The nuclei were counterstained with DAPI (blue). 400× magnification, bar = 20 μm. (**b**) The MFIs of MTDH and PTEN in the cells measured with the graphics module of Zeiss Zen software. Error bars represent the SD. (**c**) Western blotting analyses demonstrated a strong decrease in the levels of MTDH protein and a strong increase in the levels of PTEN in SKOV3/DDP cells by the sera or LY294002. The mAb-PTEN, pAb-MTDH and mAb-GAPDH were used for blotting. A total of 20 μg of protein per sample was loaded to each lane. * *P* < 0.5, **P < 0.01. NS = No significant difference

Laser confocal microscopy images showed that the MFI values of the PTEN protein significantly increased and that the MFI values of the MTDH protein significantly decreased in the SKOV3/DDP cells after treatment with the sera containing GFWE in a dose-dependent manner. The effect of the HD sera on the PTEN protein was similar to that of LY294002 (Fig. 4 a and b). Western blotting analyses demonstrated the highest MTDH protein expression and undetectable PTEN expression in SKOV3/DDP cells treated with the control sera, suggesting a stronger resistance of SKOV3/DDP cells. The observed difference between western blotting and laser confocal microscopy for detecting PTEN in the control sera-treated cells may be due to the latter being more sensitive than the former. By western blotting, the sera containing GFWE showed a similar dose-effect pattern to the inhibition of the MTDH protein and the induction of the PTEN protein by laser confocal microscopy (Fig. 4 c). These findings support earlier conclusions that MTDH overexpression and the loss of PTEN were strongly associated with cisplatin resistance, and the sera restored the cisplatin sensitivity to SKOV3/DDP cells by inhibiting MTDH and inducing PTEN.

### Sera containing GFWE enhance the colocalization of MTDH with PTEN

**Fig. 5:**
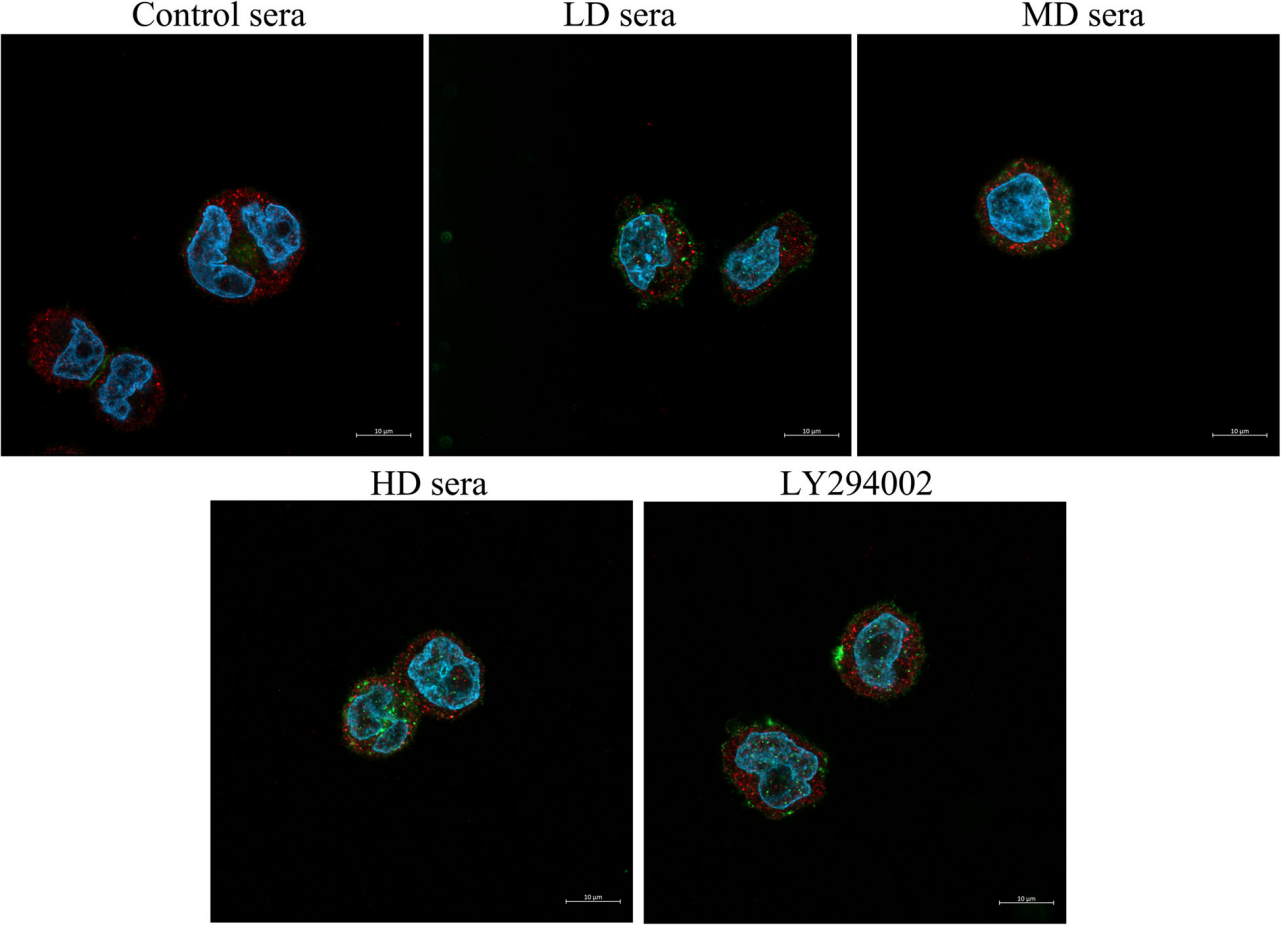
Representative images show MTDH colocalization with PTEN. All images were obtained using the 63× Plan-Apochromat oil objective of the LSM800 confocal microscope; 630× magnification, bar = 10 μm. All of the images are merged pictures of the green-colored Alexa Fluor-488 tagged the PTEN protein, the red-colored Alexa Fluor-647-tagged the MTDH protein, and the blue was the nucleus counterstained by DAPI

**Fig. 6:**
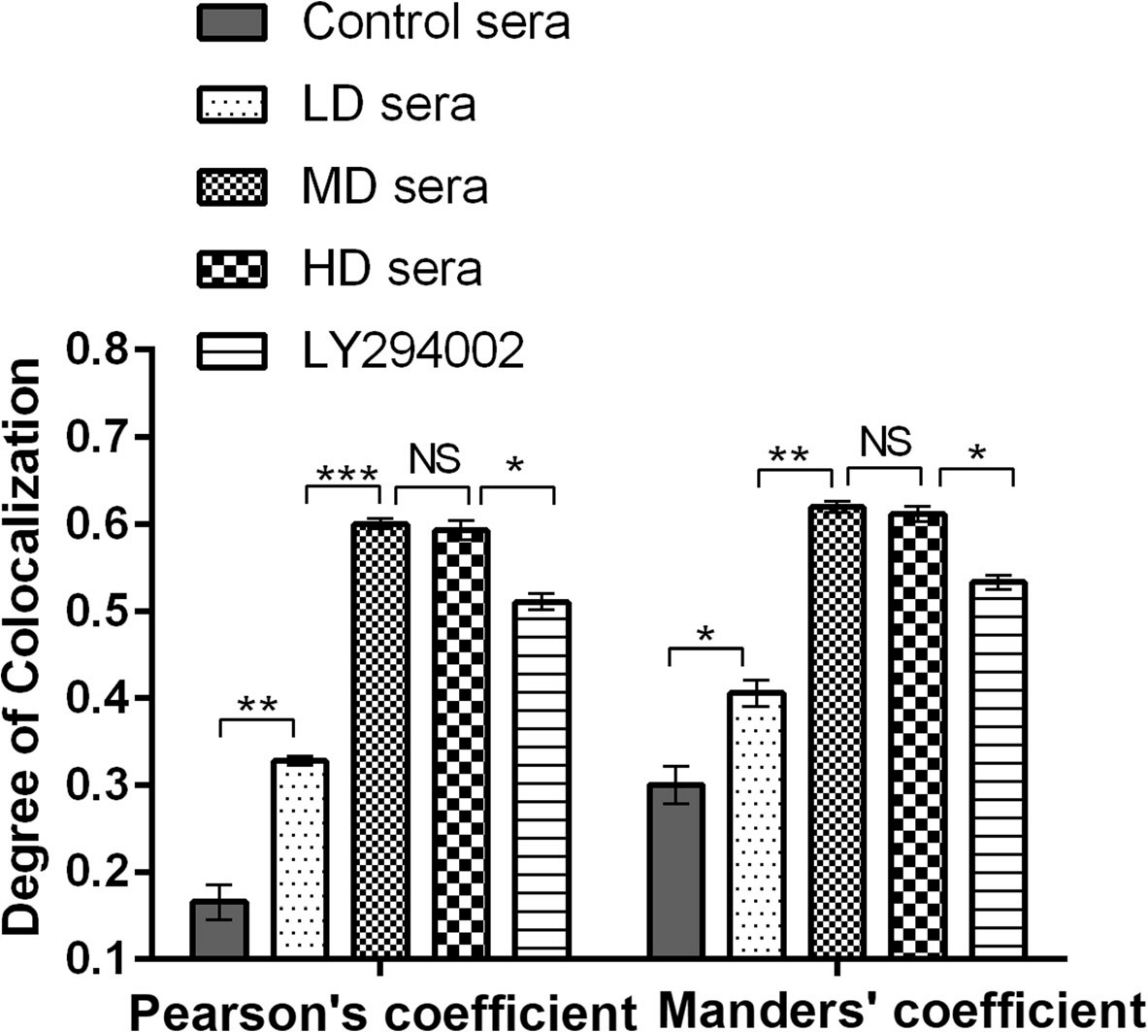
Degree of MTDH colocalization with PTEN in SKOV3/DDP cells. This bar graph represents the corresponding mean Pearson’s coefficient and Mander’s coefficient for MTDH and PTEN affected by the sera containing GFWE and LY294002. Error bars represent the SD. All calculations for Pearson’s and Mander’s coefficient have been analyzed by the confocal topography module of Zeiss Zen software. ***P < 0.001. NS = No significant difference

Combined staining of MTDH with PTEN revealed that the sera containing GFWE and LY294002 changed the protein colocalization with each other, which is most clearly visible on the larger magnification images (Fig. 5). Analyses of these images using Pearson’s coefficient and Manders’ coefficient, which generally indicate that the proteins colocalize when they are more significant than 0.5, showed that the colocalization of MTDH with PTEN occurred to a greater extent in the cells treated with the sera than in the cells treated with the blank control sera. The MD and HD sera have the highest effect on the colocalization of MTDH with PTEN, while the coefficients have no significant difference between the MD and HD sera-treated cells (*P* > 0.05, Fig. 6).

### Sera containing GFWE facilitated the interaction of MTDH with PTEN

**Fig. 7:**
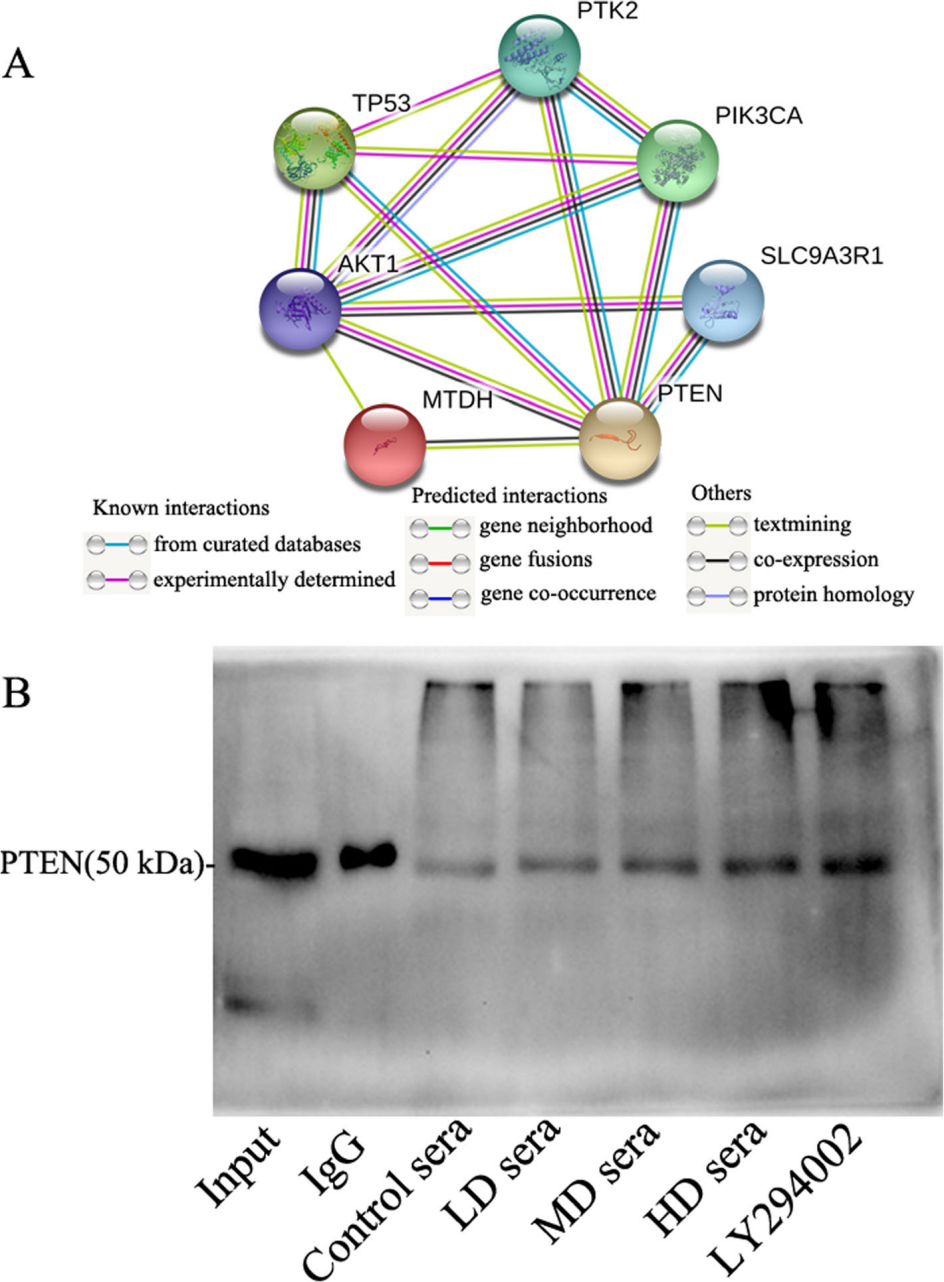
Identification and characterization of the MTDH-PTEN interaction. (**a**) Analysis of the MTDH-PTEN interaction by the STRING database. The edge indicates an interaction between the two proteins. (**b**) Interaction of PTEN with MTDH detected by co-IP. The pAb-MTDH was used for immunoprecipitation, and a rabbit IgG pAb was used as a control antibody. The mAb-PTEN was used for western blotting. The Input blot of PTEN indicates non-SureBeads-handled cell lysate to prove that there was the protein of interest in the sample to begin with

STRING database analysis indicated that there was no known direct interaction between MTDH and PTEN; only text mining has shown that there was a possible interaction between the two proteins (Fig. 7 a). To further investigate the association of MTDH with PTEN, coimmunoprecipitation was performed. PTEN was detected in immune complexes immunoprecipitated with the anti-MTDH antibody. As demonstrated in Fig. 6 c, the PTEN protein was discovered in the fractions of approximately 50 kDa, corresponding to the monomeric form of the PTEN protein and is in line with Shen’s previous results [48]. Compared with that in the control sera, the GFWE-containing sera had increased PTEN expression, while LY294002 demonstrated the highest PTEN expression in the complexes (Fig. 7 b). This experiment indicates that there is a physical interaction between MTDH and PTEN, and the interaction increased by the sera containing GFWE and LY294002, suggesting that the interaction may serve a role in chemoresistance and targeting the interaction may be helpful to restore cancer cell sensitivity to anticancer drugs.

## Discussion

Acquired resistance is common in OC, especially in advanced-stage OC, and has been defined as a failure to respond to subsequent treatment after having previously responded to first or more lines of therapy with a platin-free interval of 6 months or longer [49]. The mechanisms underlying platin chemoresistance and how resistance occurs after chemotherapy are not fully understood. The known main mechanisms of platin resistance reported to date include cancer stem cells (CSCs) [50], epithelial-to-mesenchymal transition (EMT) [51], abnormal DNA repair-related pathways [52], and the NF-κB and PI3K/AKT/mTOR signaling pathways [53]. Some of the previously reported mechanisms provide valuable targets for overcoming platin resistance in OC. Developing anticancer drugs to avoid known resistance mechanisms is an effective strategy for the management of OC. For example, sorafenib, an antiangiogenic agent, showed a statistically and clinically significant improvement in progression-free survival in women with platin-resistant OC in a phase 2 trial [54]. However, the vital ideal that researchers cannot give up is to develop targeted interventions aimed at the known resistance mechanisms and find customized strategies to assist clinicians and patients in making better-informed treatment decisions. Several studies have revealed some possible correlations between the known resistance mechanisms in cancer. Tsou SH et al. demonstrated that the activation of the NF-κB signaling pathway was partially attributable to EMT in MCF-7 cells; thus, the cells acquire stem cell-like properties, which facilitate drug resistance [55]. In another report, it was found that the doxorubicin-resistance of gastric cancer cells was related to EMT, which was induced by activating Akt and inhibiting PTEN [56]. MTDH also plays an essential role in promoting CSC accumulation [21] and inducing EMT [57]. All these observations demonstrated a strong relationship between MTDH/PTEN and known resistance mechanisms, suggesting that MTDH and PTEN are promising therapeutic targets for the chemoresistance of OC.

Traditional Chinese medicine (TCM) is a style of traditional medicine based on more than 2500 years of Chinese medical practice. TCM practitioners use herbal medicines, including Chinese classical herbal formulas, to treat or prevent health problems, whereas the biological characteristics and molecular mechanisms remain largely unknown. The use of herbal formulas differs from plant-derived drugs in standard pharmacology because it does not isolate or standardize bioactive compounds, which may result in the loss of potential essential compounds at the time of separating others. In the present study, a seropharmacology method arising recently for the pharmacological study was adopted to research TCM in vitro using medicated animal sera [58]. The essence of seropharmacology is to administer herbal formulas to experimental animals (generally rabbits or rats), followed by harvesting the animal sera after a period of time. After searching the PubMed and CNKI databases, 16 bioactive compounds were identified in the sera in the published articles. The molecular docking results confirmed that 14 of 16 compounds have higher binding activity with PTEN/MTDH compared to LY294002. Among them, gallic acid, cinnamic acid, benzoic acid, catechin, and protocatechuic acid are phenolic compounds that have been demonstrated to upregulate PTEN, p53, p21, and p27 and inhibit tumor angiogenesis and metastasis [59]. Paeonol has been shown to inhibit MTDH and induce PTEN expression in SKOV3/DDP cells in our previous study [37]. The downregulation of MTDH by polyporenic acid C was also demonstrated by other researchers [60]. In the present study, we documented that the sera containing GFWE, together with the ample evidence of the bioactive compounds, has an inhibitory effect on MTDH and an inducting effect on PTEN.

The direct interaction between MTDH and PTEN has not been reported elsewhere. However, WASH complex subunit 5 (WASHC5; also known as RTSC, SPG8, RTSC1, and KIAA0196) was reported to be coexpressed with MTDH [61] and to genetically interact with PTEN [62], suggesting that WASHC5 may interact with MTDH and PTEN. Similarly, plectin (PLEC) [63], neural precursor cell expressed developmentally downregulated 4 (NEDD4), and E3 ubiquitin-protein ligase [64] were all reported physical interactions or predicted genetic interactions from the Biological General Repository for Interaction Datasets [65], with MTDH and PTEN, respectively. These findings support the possible view that MTDH may physically interact with PTEN. In the present study, we first demonstrated that MTDH colocalizes and physically interacts with PTEN, and the colocalization and interaction statuses were changed by sera containing GFWE and LY294002. These findings in the present study provide a robust evidence base for the role of MTDH-PTEN complexes in chemoresistance, and the complexes may be a therapeutic target for cancer treatment. However, further work is required to establish the probability of the MTDH-PTEN complexes interacting with WASHC5, PLEC, or NEDD4. Another important finding from other researchers was that the loss of PTEN was associated with resistance to anti-PD-1 checkpoint blockade therapy in metastatic uterine leiomyosarcoma [66]. MTDH has also demonstrated a role in cancer immunotherapy [67]. A further study with more focus on the MTDH-PTEN complexes in cancer immunity is therefore suggested.

## Conclusions

In summary, the present study fills a gap in the literature by demonstrating that MTDH is coexpressed and physically interacts with PTEN in resistant cancer cells. GFWE restored sensitivity to cisplatin by inhibiting MTDH expression, inducing PTEN expression, and changing the interaction status of MTDH and PTEN in SKOV3/DDP cells. Our findings suggest that GFWE may potentially be used alone or in combination with cisplatin-based chemotherapy to provide a therapeutic benefit in OC after further validation. To further develop these findings, additional molecular studies in patients with OC will be required.

## Supplementary information

**Additional file 1.** Voucher numbers for the five herbs specimens.

## Data Availability

The datasets and supporting materials during the current study are available from the corresponding authors on reasonable request.

## Abbreviations

CBP: CREB-binding Protein
CSC: Cancer stem cell
EMT: Epithelial to mesenchymal transition
GFW: Guizhi Fuling Wan
GFWE: Guizhi Fuling Wan extract
MTDH: Metadherin
NEDD4: Neural precursor cell expressed, developmentally down-regulated 4, E3 ubiquitin protein ligase
P-gp: P-glycoprotein
PLEC: Plectin
PPI: Protein-protein interactions
PTEN: Phosphatase and tensin homolog
TWIST1: Twist-related protein 1
WASHC5: WASH complex subunit 5
