# Supplementary figures and images for: Overcoming cisplatin resistance by targeting the MTDH-PTEN interaction in ovarian cancer with sera derived from rats exposed to Guizhi Fuling wan extract
Source: BMC Complement Med Ther. 2020 Feb 17;20:57. doi: 10.1186/s12906-020-2825-9 (PMC7076886; doi:10.1186/s12906-020-2825-9)

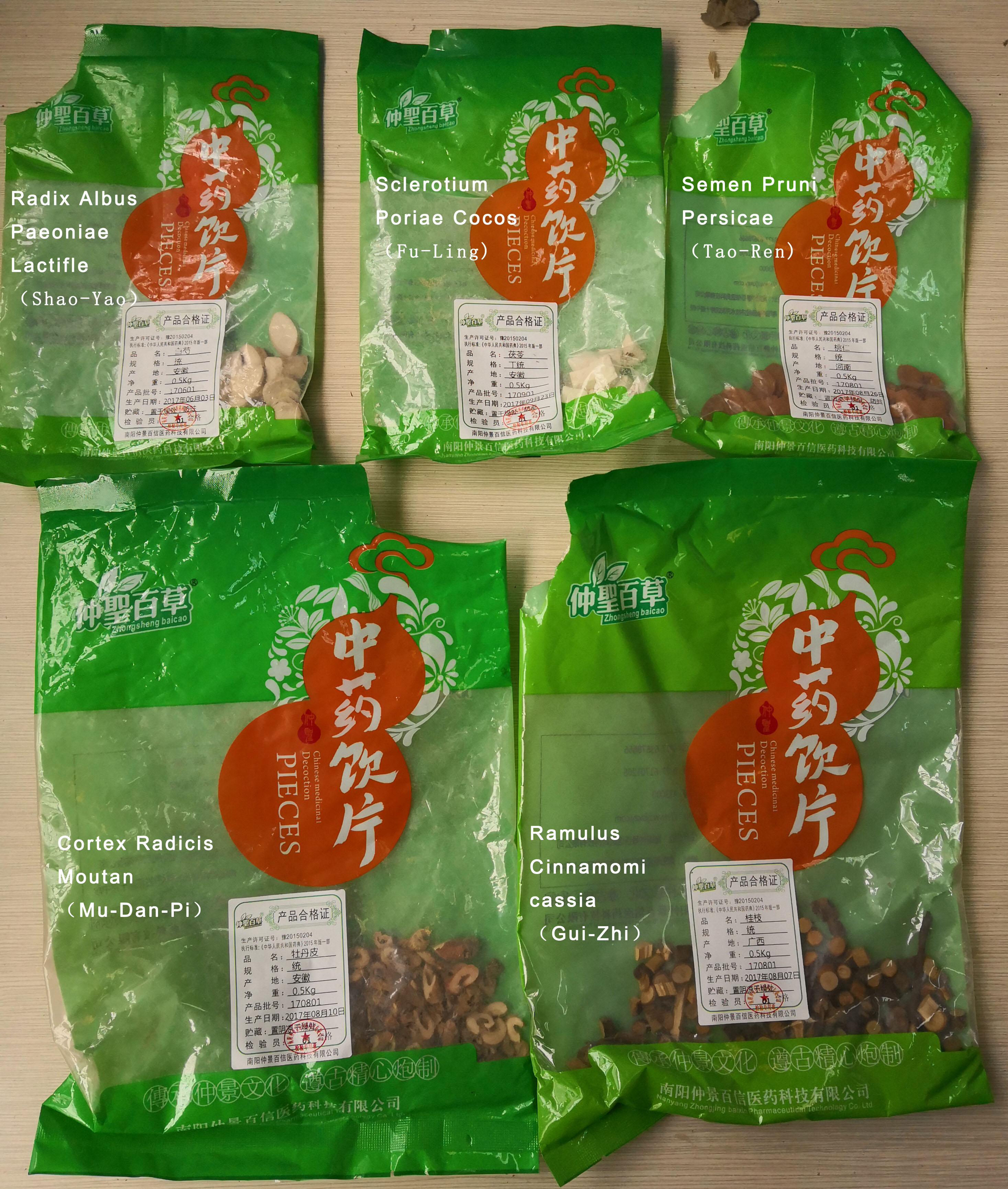

Supplement: Supplementary file 1 — Additional file 1. Voucher numbers for the five herbs specimens. [file 12906_2020_2825_MOESM1_ESM.jpg]
